# Supplementary material for: Hereditary Tyrosinemia Type 1 Mice under Continuous Nitisinone Treatment Display Remnants of an Uncorrected Liver Disease Phenotype
Source: Genes (Basel). 2023 Mar 11;14(3):693. doi: 10.3390/genes14030693 (PMC10047938; doi:10.3390/genes14030693)
Supplement: Supplementary file 1 [file genes-14-00693-s001.zip › genes-2253651-supplementary.pdf]

Supplementary Information

Hereditary tyrosinemia type 1 mice under continuous nitisinone treatment display remnants of an uncorrected liver disease phenotype

Jessie Neuckermans, Sien Lequeue, Paul Claes, Anja Heymans, Juliette H. Hughes, Haaike Colemonts-Vroninks, Lionel Marcélis, Georges Casimir, Philippe Goyens, Geert A. Martens, James A. Gallagher, Tamara Vanhaecke, George Bou-Gharios and Joery De Kock

Table S1: modulated genes per canonical pathway

Canonical Pathways	Genes
Tyrosine Degradation I	*Fah, Hgd*
FXR/RXR Activation	*Abcg8, Lpl, Saa1, Slc22a7*
Adipogenesis pathway	*Arntl, Lpl, Nr1d1, Rbp1*
Circadian Rhythm Signaling	*Arntl, Dbp, Nfil3, Nr1d1, Nr1d2*
Bupropion Degradation	*Cyp2c38, Cyp3a41b*
Asparagine Biosynthesis I	*Asns*
LXR/RXR Activation	*Abcg8, Lpl, Saa1*
Acetone Degradation I (to Methylglyoxal)	*Cyp2c38, Cyp3a41b*
Estrogen Biosynthesis	*Cyp2c38, Cyp3a41b*
Melatonin Degradation I	*Cyp2c38, Cyp3a41b*
Nicotine Degradation III	*Cyp2c38, Cyp3a41b*
Retinol Biosynthesis	*Lpl, Rbp1*
Superpathway of Melatonin Degradation	*Cyp2c38, Cyp3a41b*
Oxidative Ethanol Degradation III	*Acss3, Cyp2c38*
Aryl Hydrocarbon Receptor Signaling	*Cyp2c38, Cyp3a41b, Nqo1*
Nicotine Degradation II	*Cyp2c38, Cyp3a41b*
Retinoate Biosynthesis II	*Rbp1*
SPINK1 General Cancer Pathway	*Mt1, Mt2*
PXR/RXR Activation	*Cyp2c38, Cyp3a41b*
Catecholamine Biosynthesis	*Ddc*
Acetate Conversion to Acetyl-CoA	*Acss3*
Acute Phase Response Signaling	*Rbp1, Saa1, Saa3*
Hepatic Cholestasis	*Abcg8, Cyp3a41b, Slc22a7*
Serotonin and Melatonin Biosynthesis	*Ddc*

Table S2: Differential gene expression in liver tissue of FAH+NTBC *versus* AKU+NTBC mice with 2-fold cut-off

Gene symbol	Gene name	F.C.
Abcg8	ATP binding cassette subfamily G member 8	2.0
Acss3	Acyl-CoA Synthetase Short chain Family member 3	-2.3
Arntl	Aryl hydrocarbon receptor nuclear translocator-like protein	-2.6
Asns	Asparagine synthetase	3.7
Cxcl1	Chemokine ligand 1	4.3
Cyp2c38	Cytochrome P450, family 2, subfamily c, polypeptide 38	-2.2
Cyp3a41b	Cytochrome P450, family 3, subfamily a, polypeptide 1b	-2.6
Dbp	D-box binding protein	5.2
Ddc	Dopa Decarboxylase	-2.3
Egr1	Early growth response protein 1	3.6
Elovl3	Elongation of Very Long Chain Fatty acid protein 3	-2.9
Fah	Fumarylacetoacetate hydrolase	-9.6
Fitm1	Fat Storage Inducing Transmembrane protein 1	-2.2
Hgd	Homogentisate dioxygenase	65.3
Lp1	Lipid transfer protein 1	2.0
Moxd1	Monooxygenase dopamine beta-hydroxylase like 1	8.5
Mt1	Metallothionein 1	7.3
Mt2	Metallothionein 2	6.7
Nfil3	Nuclear factor, interleukin 3 regulated	-2.1
Nqo1	NAD(P)H quinone oxidoreductase 1	2.0
Nr1d1	Nuclear orphan receptor group D member 1	3.0
Nr1d2	Nuclear orphan receptor group D member 2	2.5
Rbp1	Retinol-binding protein 1	2.2
Saa1	Serum amyloid A1	3.1
Saa2	Serum amyloid A2	6.6
Saa3	Serum amyloid A3	2.2
Slc22a7	Solute carrier Family 22 member 7	-2.1
